# A novel fixel-based approach for resolving neonatal white matter microstructure from clinical diffusion MRI

**DOI:** 10.3389/fnins.2026.1830684

**Published:** 2026-06-05

**Authors:** Benjamin T. Newman, John D. Van Horn, T. Jason Druzgal, Meghan H. Puglia

**Affiliations:** 1 Department of Radiology and Medical Imaging, University of Virginia, Charlottesville, VA, United States; 2 Department of Psychology, University of Virginia, Charlottesville, VA, United States; 3 School of Data Science, University of Virginia, Charlottesville, VA, United States; 4 Department of Neurology, University of Virginia, Charlottesville, VA, United States

**Keywords:** clinical neuroimaging, diffusion MRI, fixel-based analysis, neonatal brain development, preterm birth, white matter microstructure

## Abstract

**Introduction:**

Preterm birth is a major risk factor for disrupted brain development and subsequent neurodevelopmental disorders, yet the underlying mechanisms remain poorly understood. Further, typical neuroimaging analyses are particularly challenging in the neonatal brain: data is frequently low quality, and a lack of cellular development violates the assumptions relied on by many commonly-used techniques. In this study, we develop and present an advanced diffusion magnetic resonance imaging method to examine the microstructural organization of white matter in a clinically-acquired cohort of premature neonates.

**Methods:**

Using a novel approach that resolves multiple tissue compartments within the brain, we provide highly detailed orientation and quantification of white matter fibers and tissue signal fraction. We also utilize a series of automated segmentation algorithms to identify and measure these metrics across key tracts and subcortical regions. We investigate how these measures relate to postmenstrual age, as well as to clinical factors reflecting neonatal illness severity.

**Results:**

We report successful segmentation and reconstruction of numerous white matter tracts throughout the neonatal brain. We further demonstrate the utility and functionality of microstructural analysis in a variety of pathologies commonly encountered in the neonatal clinical environment. Our results demonstrate tract-specific developmental trajectories, with early-maturing pathways showing higher microstructural organization. Exploratory analyses suggest that neonatal illness severity has modest, tissue-specific associations with microstructural properties.

**Discussion:**

This work demonstrates that advanced microstructural imaging methods can extract meaningful white matter measurements from clinically-acquired scans, providing a practical framework for studying neonatal brain development in real-world hospital settings. These metrics are able to be calculated at extremely young ages, potentially allowing non-invasive study of vulnerable populations before detailed behavioral or neurological assessments are feasible.

## 1 Introduction

Preterm birth, defined as delivery before 37 weeks' gestation, affects roughly 10% of births in the United States (Centers for Disease Control Prevention, 2022) and is a leading contributor of long-term neurodevelopmental disorders (Schieve et al., 2016). Infants born preterm are at heightened risk for a broad range of cognitive, motor, and socio-emotional impairments, including learning disabilities, attention-deficit/hyperactivity disorder, and autism spectrum disorder (Johnson, 2007; Lindström et al., 2011; Moreira et al., 2014; Agrawal et al., 2018; Brydges et al., 2018; Crump et al., 2021). Despite this elevated risk, the mechanisms linking preterm birth to later neurodevelopmental outcomes are not fully understood.

A hallmark of preterm brain injury is disruption to white matter (Ball et al., 2012; Guo et al., 2017; Hinojosa-Rodríguez et al., 2017), which is particularly vulnerable during the late gestational period when axonal growth, oligodendrocyte maturation, and myelination are actively occurring (Ortinau and Neil, 2015; Bouyssi-Kobar et al., 2016; Volpe, 2019). Diffusion MRI (dMRI) has emerged as a powerful tool for quantifying white matter microstructural features at the subcellular level. This technique measures the Brownian movement of water molecules within brain tissue, which is influenced by cellular structures such as axons, myelin, and surrounding extracellular space (Le Bihan and Iima, 2015). From these diffusion patterns, researchers can derive measures that reflect different aspects of tissue organization. For example, fiber density estimates the relative amount of axonal density and fiber diameter within a white matter pathway, providing an indicator of the number or packing of fibers in a tract (Raffelt et al., 2017). Three-Tissue Constrained Spherical Deconvolution (3T-CSD) signal fractions reflects the proportion of the diffusion signal arising from different tissue compartments, including anisotropic white matter-like signal, and isotropic gray matter-like and cerebrospinal fluid (CSF)-like components. The anisotropic white matter–like signal reflects directionally-constrained diffusion arising from both intra-axonal and supporting glial cells involved in myelination, while the isotropic components capture more adirectional diffusion in larger intracellular compartments and bodies or extracellular unrestricted diffusion (Frigo et al., 2020). These microstructural compartments have been shown to vary across the lifespan (Newman et al., 2025c) and developmental period (Newman et al., 2023), and to correlate with pathological changes in cellular integrity (Dhollander et al., 2017; Kelly et al., 2022; Newman et al., 2025a). Together, these measures provide complementary information about the structural integrity and organization of neural pathways and can help characterize early brain development.

Prior diffusion studies in preterm neonates have consistently demonstrated delayed maturation and altered connectivity in key white matter tracts. These studies have identified microstructural differences in major projection and association pathways, including the corticospinal tracts, thalamocortical tracts, corpus callosum, and superior longitudinal fasciculi (Ball et al., 2012, 2013; Braga et al., 2015; Guo et al., 2017; Pannek et al., 2018; Pecheva et al., 2019) that have been linked to poorer motor, language, and cognitive outcomes in early childhood (Spittle et al., 2011; Keunen et al., 2017; Salvan et al., 2017). Collectively, these findings suggest that diffusion MRI can detect subtle microstructural disruptions in white matter organization that may precede overt neurological or developmental impairments, offering a window into the early neural substrates of preterm risk.

Despite these prior successes, neonatal brains pose unique challenges for traditional diffusion imaging: myelin is largely undeveloped, extracellular water is abundant, and directional diffusion signals are weak and easily obscured (Dubois et al., 2021). Clinical imaging settings introduce additional limitations, including low b-values and limited angular resolution, which result in lower image detail and fewer angles of measurement, further complicating reliable microstructural estimation (Vos et al., 2016).

To address these challenges in neonatal dMRI analyses (Verschuur et al., 2025), we adapt a novel single-shell three-tissue constrained spherical deconvolution (SS3T-CSD) approach for low b-value clinical data, incorporating a highly restricted white matter response function to quantify both intra-axonal and extra-axonal diffusion signals in a cohort of preterm neonates. This method allows for refined estimation of fiber orientation distributions, detailed fiber tracking, and calculation of advanced fixel-based metrics of white matter integrity. Using this approach, we examine how white matter microstructure relates to postmenstrual age to better understand early brain maturation following preterm birth, as well as how it relates to clinical factors and neonatal outcomes to identify potential influences on long-term neurodevelopment. We also demonstrate the utility of the method in atypical neuroanatomy, such as neonates with non-standard geometries, and provide a preterm neonatal fiber orientation distribution template to facilitate future stereotaxic alignment and fixel-based analyses.

## 2 Materials and methods

### 2.1 Participants

Participants were drawn from an ongoing prospective cohort study of infants admitted to the University of Virginia Neonatal Intensive Care Unit (NICU). The study protocol was approved by the University of Virginia Institutional Review Board for Health Sciences Research (HSR210330). Written informed consent was obtained from the parents or legal guardians of all participants prior to participation.

For the present analysis, we included 54 infants from this cohort who underwent clinically-acquired dMRI during routine care. All scans were acquired on a Siemens Prisma 3T scanner at the University of Virginia Hospital using clinical protocols. Acquisition parameters for each scan are detailed in Supplementary Table S1. Forty-nine infants passed manual quality control checks for excessive motion or distortion, and a further six were removed during the analysis process for poor masking or anatomical segmentation. The final analytic sample consisted of 43 infants.

### 2.2 Diffusion image preprocessing

**Figure 1:**
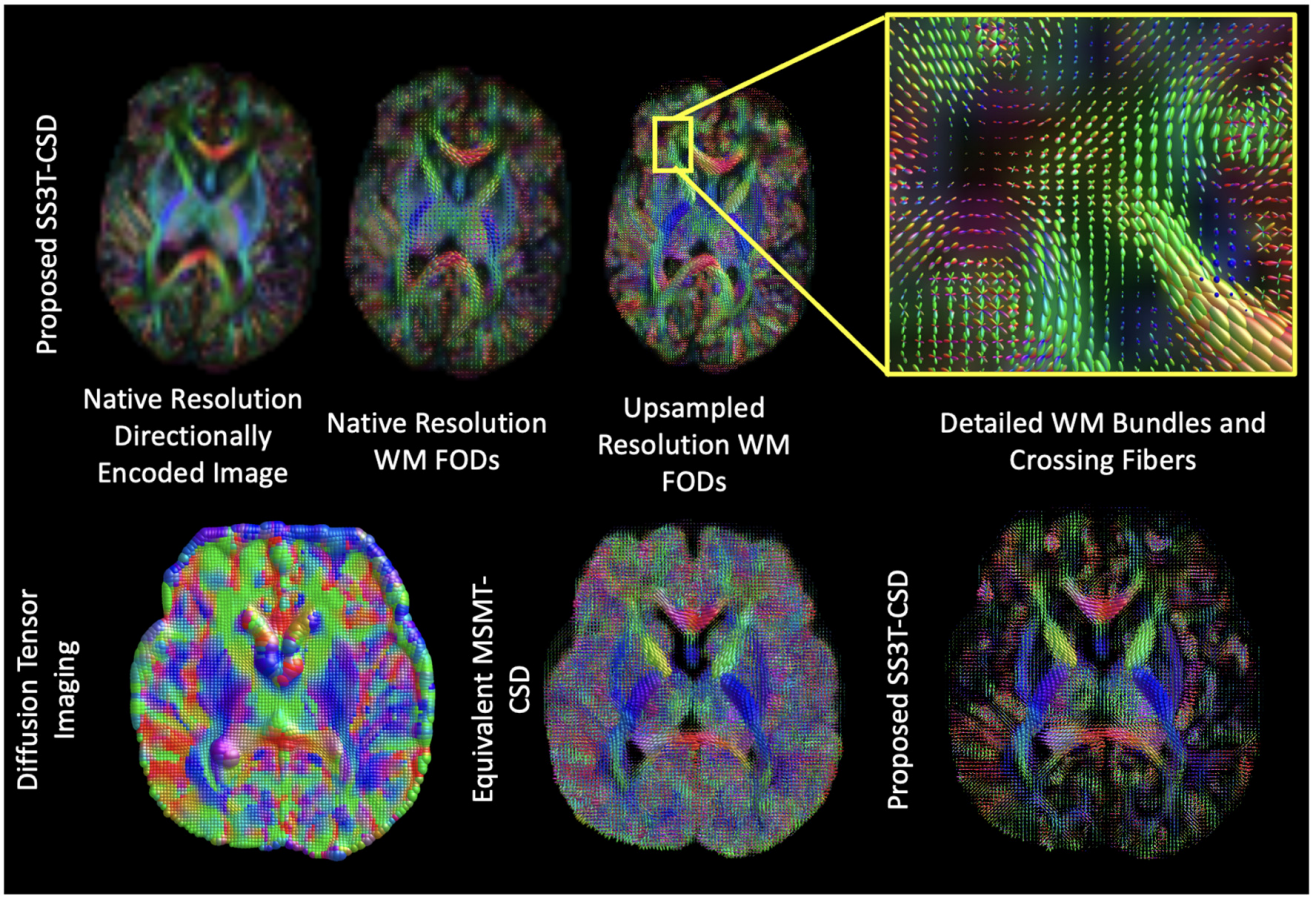
Comparison of white matter fiber orientation distribution reconstruction across preprocessing and modeling approaches. The preprocessing techniques presented in this study improve white matter fiber orientation distribution (FOD) estimation and enable visualization of crossing fibers in neonatal clinical diffusion data **(top row)**. For illustrative comparison, alternative diffusion models are shown in a separate subject **(bottom row)**. Diffusion tensor imaging (DTI) is limited in resolving crossing fibers, while multi-shell multi-tissue constrained spherical deconvolution (MSMT-CSD), when applied to these single-shell data, incompletely separates tissue compartments and underestimates white matter-like signal. In contrast, SS3T-CSD resolves white matter, gray matter-like, and CSF-like compartments from single-shell data typically utilized in clinical contexts without requiring research quality multi-shell acquisitions.

DICOM images were obtained directly from the University of Virginia Health System's Picture Archiving and Communication System (PACS). Images were anonymized and organized using dcm2niix for nifty conversion and gradient table extraction (Rorden, 2025). Masking and brain extraction was performed directly on the mean b = 0 image using a specially adapted version of the Rapid Automatic Tissue Segmentation tool (Oguz et al., 2014). The brain extraction algorithm was initialized using an estimate of the 50th percentile brain volume at birth 340,000 mm^3^ as reported in the literature (Holland et al., 2014). Masks were then visually inspected and, if brain was not sufficiently segmented, the initialization volume was reduced in a step-wise manner by 10,000 mm^3^ with visual inspection after each run until all non-brain tissue was successfully removed. This step-wise procedure provided superior results for the most extremely preterm infants. All diffusion images were then denoised (Veraart et al., 2016), Gibbs rings were removed (Kellner et al., 2016), and motion correction with outlier replacement was performed according to established preprocessing protocols (Andersson et al., 2016). No reverse-phase images were collected as part of the clinical process, however *eddy* was utilized for motion correction and could be used with *topup* for distortion correction if the appropriate acquisition was collected (Jenkinson et al., 2012). Images were then uniformly upsampled to a spatial resolution of 1 × 1 × 1 mm^3^ to minimize partial voluming effects and enforce isotropic voxels to harmonize different clinical acquisitions (Greenspan, 2009; Newman et al., 2020a). This preprocessing step also improves the resolution and interpretability of fiber orientation distributions, enabling clearer identification of crossing fibers (Figure 1).

#### 2.2.1 Constrained spherical deconvolution application

Following preprocessing, the diffusion images were analyzed using only the b = 0 and the b = 1,000 s/mm^2^ shell. This may initially appear counterintuitive, especially as the Developing Human Connectome Project acquired an outer shell of b = 3,000 s/mm^2^ (Pannek et al., 2012) and when compared to typical adult and adolescent dMRI imaging specifications. It has also been reported that diffusion tensor imaging (DTI) measured fractional anisotropy (FA) values are stable in neonates up to b = 3,000 s/mm^2^ (Dudink et al., 2008). However, the lower b-value shell provides superior signal-to-noise ratio and ability to resolve poorly myelinated axonal fibers compared to higher b-value shells where the already low directional white matter signal can be overwhelmed by noise. This lower b-value shell does include significantly greater isotropic and extracellular signal, however we have implemented several innovative approaches to identify and remove isotropic signal. This is also a far more typical acquisition for clinical purposes, where speed is prioritized to reduce the time a vulnerable neonate must be physically in the scanner, and thus our results will be more widely applicable to both retrospective and prospective studies.

A further issue that must be confronted in neonatal dMRI, specifically when applying CSD-based models, is the inhomogeneity of white matter fibers across the developing neonatal brain. CSD-based techniques assume that the white matter fiber orientation distribution function can be obtained by deconvolving within each voxel in the brain a single shared common single fiber response function (Tournier et al., 2007; Jeurissen et al., 2014). This response function is typically not defined *a priori*, and is instead derived from a group-wise average value extracted from “single fiber voxels,” which are typically voxels with the highest FA (Dhollander et al., 2016). In the neonatal milieu, the assumptions underlying both CSD and response function definition can be themselves problematic and lead to poor detection and deconvolution of white matter throughout the brain. First, inhomogeneities between white matter fibers are a known issue in adult brains affecting CSD performance (Parker et al., 2013), and neonates display even greater degrees of temporal and spatial white matter inhomogeneities (Bastiani et al., 2019). FA has been found to be far lower throughout the neonatal brain compared to the adult brain (Dudink et al., 2008; Guo et al., 2017; Toselli et al., 2017), which may bias the white matter response function.

Secondly, and a related problem, is that in the absence of well-myelinated axonal fibers, there is a generalized high level of extracellular fluid and isotropic diffusion signal (indicated by increased mean diffusivity) throughout the neonatal white matter skeleton and brain (Bartha et al., 2007; Kimpton et al., 2021). A third issue is that signal from isotropic fluid remains relatively magnified at b = 1,000 s/mm^2^, with intra-axonal signal having a similar intensity to some extra-axonal signals (Cihangiroglu et al., 2011; Jeurissen et al., 2014). These extra-axonal signals have traditionally been heavily restrictive on fiber tracking and make detection and resolution of crossing fibers difficult (Makki and Hagmann, 2017). While prospective studies in more mature populations can simply acquire higher b-value shells to address this problem, for reasons both clinical, retrospective, and anatomical, an effective methodology for studying the neonatal brain must address these weaknesses at low b-values.

Our approach addresses these issues with several innovations. The workhorse at the center of this approach is single shell 3-tissue constrained spherical deconvolution (SS3T-CSD; Dhollander and Connelly, 2016a; Dhollander et al., 2018). While other CSD and diffusion models generally perform a single fitting step to describe the diffusion signal, SS3T-CSD instead iterates between two microstructural tissue compartments for three complete cycles. For each iteration, the algorithm fits two response functions to the observed signal, which is always assumed to be isotropic in nature. First, extracellular (CSF-like) signal is separated from intracellular signal by fitting both CSF and gray matter-like response functions to the observed signal. Then, the white matter response function is separated from the remaining intracellular (gray matter-like) signal. This “cleans” the raw diffusion signal with each iteration, identifying directional white matter-like signal and removing isotropic signal that can occlude the relatively weak neonatal white matter signal. A further benefit of the SS3T-CSD approach to both clinical and neonatal data is that the contribution of b = 0 images is weighted to 10% of the total signal, rather than an equal weight contributed from all shells as in standard multi-shell multi-tissue CSD (MSMT-CSD; Jeurissen et al., 2014). This alteration of weights greatly assists in identifying and removing the extracellular CSF-like fluid that is present throughout the neonatal brain, as CSF-like fluid both experiences the highest intensity at b = 0 and the greatest signal drop-off as b-values increase. Minimizing the b = 0 contribution to total signal thus acts to suppress extracellular isotropic signal, while SS3T-CSD iteratively identifies and separates this signal from intracellular signal, improving white matter resolution. An example of single compartment DTI and MSMT-CSD derived white matter fiber orientation distribution (FOD) is displayed on the bottom of Figure 1, illustrating the ability of SS3T-CSD to derive improved white matter detail and separation from isotropic signal.

In order to be effective, however, SS3T-CSD must also correctly identify white matter signal, despite the inhomogeneities across white matter fibers in the neonatal brain. To combat this, we have defined our white matter response function *a priori* using the Dhollander et al. (2019) algorithm. This approach defines an artificial “extreme” single fiber response function using zonal spherical harmonics and further penalizes the presence of extracellular fluid in locating and defining a cohort-specific white matter response function. This approach was demonstrated to significantly improve white matter identification and detection, greatly increasing contrast between white and gray matter in neonates and infants (Dhollander et al., 2019). Response functions for white matter, gray matter, and CSF were defined in each subject in this study, then averaged together to generate mean response functions for use in SS3T-CSD. Both the response function selection algorithm and SS3T-CSD are available from MRtrix3Tissue (https://3tissue.github.io/), a fork of MRtrix3 (Tournier et al., 2019).

#### 2.2.2 Post-processing and microstructural analysis

**Figure 2:**
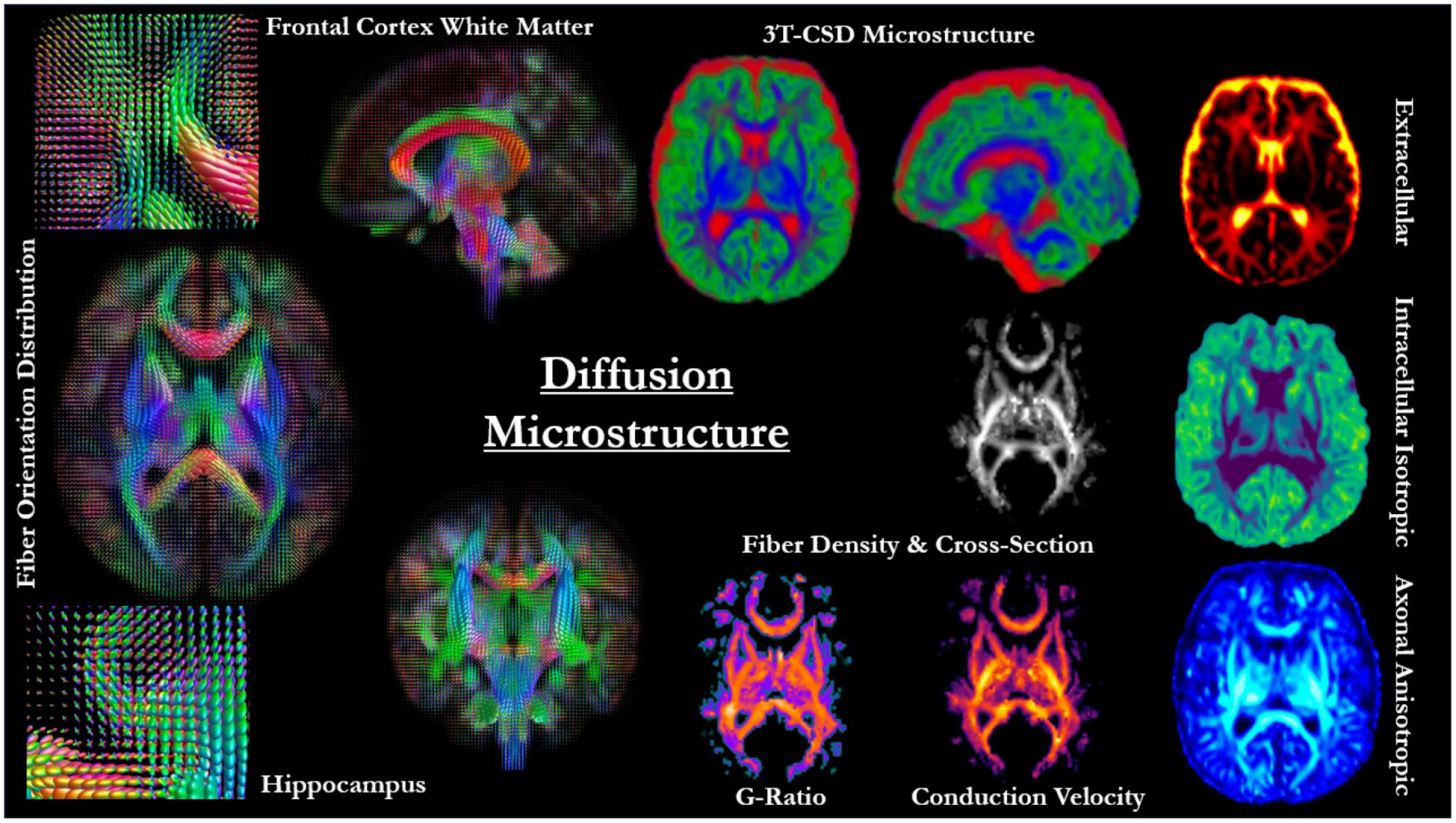
Representative output microstructure maps. Using techniques described in this study, we are able to detect and resolve axonal white matter fibers throughout the brain and generate a wide variety of diffusion microstructure maps from both 3T-CSD and fixel-based techniques. This figure also demonstrates how, with an available T1- and T2-weighted image, additional microstructural metrics such as aggregate g-ratio and aggregate conduction velocity can be calculated.

Processing each subject with SS3T-CSD yielded a FOD for white matter, gray matter, and CSF. The intensities of these FODs were then log normalized using the *mtnormalise* function in MRtrix3 to correct for residual intensity inhomogeneities (Dhollander et al., 2021) and microstructure signal fraction maps created by summing the coefficients of each white matter, gray matter, and CSF FODs within each voxel to equal 1 (Newman et al., 2020b). Representative output maps from a neonatal subject are shown in Figure 2. The normalized white matter FODs from subjects without severe anatomical deficits were used to generate a group-wise mean population template for preterm neonates using the population_template command. Fixel-based fiber density was calculated for each subject according to established methods (Raffelt et al., 2017).

#### 2.2.3 Whole brain automatic segmentation and tractography

**Figure 3:**
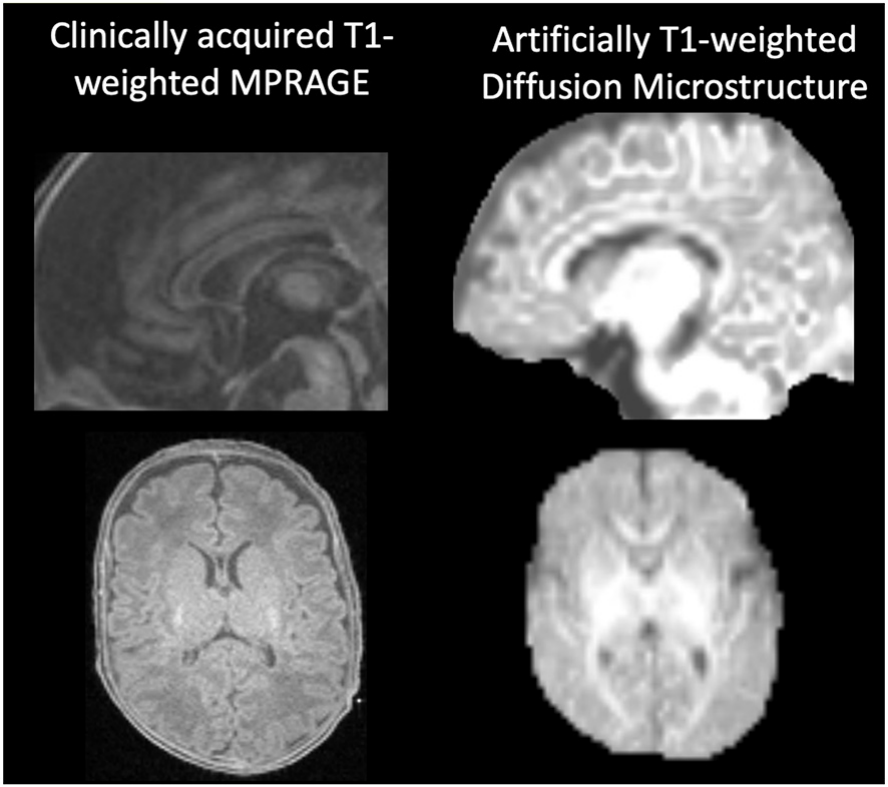
Comparison of clinically acquired T1-weighted MPRAGE and artificial contrast corrected diffusion microstructure map. Artificial maps with adult-like T1-weighted contrast were generated by re-weighing CSF, white matter, and gray matter signal fraction maps to facilitate the use of established intensity-based segmentation techniques.

**Figure 4:**
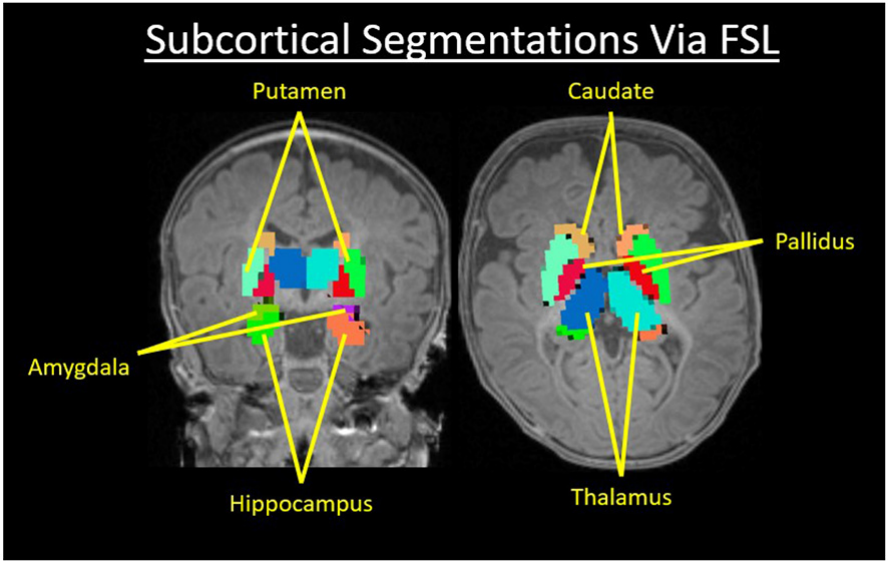
Subcortical structures segmented from artificially generated T1-weighted contrast using 3T-CSD diffusion microstructure maps. Using FSL's FIRST and FAST automated segmentation algorithms, seven bilateral subcortical structures (including the Nucleus Accumbens, not labeled above) were able to be accurately segmented across participants.

Automatic brain segmentation and parcellation is a priority for objective evaluation of subcortical structures. This process has been considered highly challenging in neonates and infants due to inverted T1w contrast between gray and white matter, with higher extracellular fluid levels in white matter causing these areas to appear darker than gray matter (Zhang et al., 2019). Automated contrast-based methods or even more advanced machine learning based methods trained on older individuals frequently struggle. A recent series of open challenges at the International Conference on Medical Image Computing and Computer Assisted Intervention (MICCAI) has attempted to address this problem in 6-month old infants (Wang et al., 2019; Sun et al., 2021). The Developing Human Connectome Project has implemented computationally based segmentation models; however, these were performed and tested on T1w and T2w images with far higher quality and collected using specialized coils that are not available in clinical practice (Hughes et al., 2017; Makropoulos et al., 2018). Further issues arise when considering that clinically collected T1w images are frequently differentially sized and positioned compared to dMRI acquisitions, with much wider FOV and resolutions that make co-registration challenging. To address these challenges and in order to employ algorithms and methods with whole lifespan translational benefits, we applied weighted contrast to the diffusion signal fraction maps to generate subject specific images with adult-like T1w contrast (Figure 3). This method has previously been demonstrated to provide similar distributions of intensity to T1w MPRAGE images in healthy adult subjects (Dhollander and Connelly, 2016b). Relative contrast was selected based on experimentally reported peak values for each tissue type, with weights of 95, 60, and 25 for white matter, gray matter, and CSF, respectively, based off of previously published results (Song et al., 2026). Each tissue compartment was multiplied on a voxel-wise basis by these weights and then summed together to generate the artificial T1w image, which generated typical T1w contrast patterns (Figure 3). The resulting image was then segmented into white matter, cortical gray matter, and subcortical gray matter using FSL's *FIRST* and *FAST* hidden Markov, Bayesian, and expectation-maximization algorithms including the average b = 0 image as T2-weighted contrast and implemented in the *5ttgen* MRtrix3 function (Zhang et al., 2002; Smith et al., 2004, 2012; Patenaude et al., 2011). Individual subcortical structures (Figure 4) were obtained from FIRST and FAST surface segmentations. Visual inspection confirmed correct subcortical structure segmentation using known anatomical landmarks including the presence of the internal capsule and the correct relative positioning of subcortical structures (i.e., the amygdala placed anterior and superior of the hippocampus; Figure 4).

**Figure 5:**
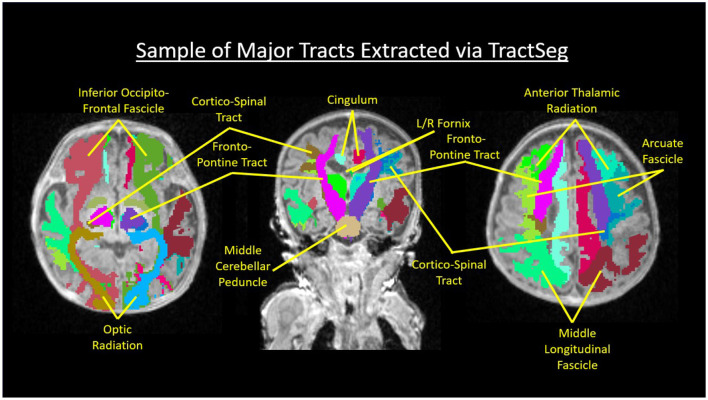
Major white matter tracts identified and extracted from neonatal FODs using the TractSeg neural network. Resolution of white matter FODs is sensitive and detailed to define and extract richly detailed maps of major white matter tracts and fasciculi.

The resulting five Tissue Type (5tt) image was then used to perform Anatomically Constrained Tractography (ACT) using the probabilistic iFOD2 algorithm to dynamically seed 10,000,000 tracts to ensure adequate reconstruction of white matter fiber populations. The 5tt image when used in ACT constrains tracts to voxels identified as white matter with maximum curvature of 45° and minimum length of 2 mm to reflect the smaller spatial scale of the neonatal brain while avoiding spurious short streamlines driven by noise. Spherical informed filtering of tractograms (SIFT) was used to reduce the number of tracts to 1,000,000 based on underlying white matter-FOD lobe integrals (Smith et al., 2013). Additionally, for more accurate segmentation of specific white matter tracts, each subject's white matter FODs were converted to spherical harmonic peaks using the *sh2peak* command in MRtrix3 (Jeurissen et al., 2013) in order to be used in the TractSeg convolutional neural network-based segmentation approach (Wasserthal et al., 2018). TractSeg was implemented on the GPU architecture maintained by University of Virginia Research Computing and used the most recently-released set of training weights to segment each subject's white matter into up to 72 tract bundles (Figure 5). Fixel-based fiber density, and 3T-CSD white matter-like, gray matter-like, and CSF-like microstructure signal fractions were averaged across all voxels in each whole brain region of interest (ROI), subcortical gray matter region, and tract bundle in each subject for statistical analysis.

### 2.3 Statistical analysis

Planned analyses were preregistered (aspredicted.org, #276926) prior to data inspection. All statistical analyses were conducted in R version 4.3.1 (R Core Team, 2020) using the lme4 (Bates et al., 2015) and lmerTest (Kuznetsova et al., 2017) packages to assess fiber density in white matter tracts and tissue signal fraction in subcortical structures.

Tracts and structures with >50% zero values across subjects were excluded prior to analysis. This pre-registered threshold was selected to exclude tracts with insufficient data for reliable estimation. These included corpus callosum regions and commissures (anterior commissure, rostrum, rostral body, anterior midbody), the fornix, inferior cerebellar peduncle, striatal–cortical tracts (striato-premotor, striato-fronto-orbital, striato-precentral, striato-postcentral), superior longitudinal fascicles (I–III), thalamo-premotor and thalamo-postcentral tracts, the superior thalamic radiation, and the uncinate fascicle. These tracts were not present in all subjects due to neurological diagnoses.

Remaining tracts were classified according to established developmental trajectories (Ball et al., 2013; Braga et al., 2015). Early-maturing tracts included cerebellar peduncles, corticospinal pathways, thalamo-cortical tracts, optic radiation, posterior corpus callosum regions, and pontine projections. Later-developing association tracts included superior, middle, and inferior longitudinal fascicles, the arcuate fascicle, inferior occipito-frontal fascicle, cingulum, striatal–cortical projections, anterior thalamic radiation, and the genu of the corpus callosum.

Subjects with zero values in >25% of the remaining tracts (*n* = 6) or subcortical structures (*n* = 3) were excluded. This pre-registered threshold was selected to exclude subjects with insufficient data for reliable estimation. Outlier values (>3 median absolute deviations above the median) were also removed, accounting for 13.3% of tract data points and 9.6% of subcortical structure data points.

Linear mixed-effects models were fit using restricted maximum likelihood (REML), with significance of fixed effects assessed via Satterthwaite's approximations for degrees of freedom. Model diagnostics, including residual distributions, random-effects structure, and singularity checks were performed to ensure model appropriateness.

All models included random effects for subjects to account for repeated measurements. For white matter tract fiber density, an additional random effect allowed each tract to vary within subject, as ANOVA comparisons confirmed that including tract-level variability significantly improved model fit. For tissue signal fraction in subcortical structures, only subject-level random effects were included because structure-level random effects exhibited low variance and resulted in singular model fits. Residual inspection further indicated that zero values distorted model estimation; therefore, remaining zero values were excluded prior to model fitting. Where possible, the same random-effects structure was used across analyses for comparability, with deviations due to singularity noted. All models included mean-centered postmenstrual age at MRI (days), mean-centered gestational age at birth (days), and sex as covariates.

Finally, to evaluate robustness of thresholding decisions, sensitivity analyses were conducted using alternative tract (>40% and >60% zero values) and subject (>15% and >35% zero values) exclusion thresholds. Primary statistical models were re-estimated under these conditions.

#### 2.3.1 Description of white matter microstructure

Descriptive statistics (mean and standard deviation) were computed for fiber density by tract and tissue signal fraction by subcortical structure to characterize the distribution, central tendency, and variability of tract-specific fiber density and tissue fraction metrics across the cohort. As a methodological validation step, we evaluated whether fiber density values reflected established neonatal white matter maturation patterns by comparing early-maturing projection and commissural tracts with later-developing association tracts. These tract classifications are defined *a priori* based on prior developmental literature (Ball et al., 2013; Braga et al., 2015) and therefore serve as a consistency check rather than independent validation of the approach. Mean fiber density values were compared between these tract classes using Welch's two-sample *t*-test. Tissue signal fractions were evaluated using a repeated-measures ANOVA with tissue type (CSF, gray matter, white matter) as a within-subject factor, followed by Bonferroni-corrected paired comparisons to assess expected tissue-specific distributions.

#### 2.3.2 Maturation of white matter microstructure

To test the hypothesis that fiber density in early-maturing tracts would increase with postmenstrual age and later-developing association tracts would show lower fiber density at comparable postmenstrual age, we included the interaction between postmenstrual age and tract classification (early vs. late) as a fixed effect in a linear mixed-effects model. The purpose of this analysis is to provide independent support for the developmental sensitivity of the model. Because eight subjects had postmenstrual age values of approximately 1 year or greater (range 363–1,459 days), a complementary model was performed in a restricted sample of younger participants (postmenstrual age < 360 days, range: 239–307) to ensure that these relative outliers did not drive observed effects.

#### 2.3.3 Maturation of tissue fractions

To test the hypothesis that subcortical gray matter fraction increases and CSF fraction decreases with postmenstrual age, we included the interaction between postmenstrual age and tissue type (CSF, gray matter, white matter) as a fixed effect in a linear mixed-effects model. Three subjects had postmenstrual age values of approximately 1 year or greater (range 363–370 days). As such, a complementary model was restricted to younger participants (postmenstrual age < 360 days, range: 253–303) to ensure these relative outliers did not drive results.

#### 2.3.4 Exploratory clinical correlates

In exploratory analyses, we examined whether greater illness severity was associated with lower fiber density and higher CSF fraction. Clinical data were extracted from the University of Virginia Health System electronic health record via the Caboodle enterprise data warehouse (Epic Systems, Madison, WI). Illness severity was characterized using the Heart Rate Observation (HeRO; Fairchild and Aschner, 2012), Neonatal Sequential Organ Failure Assessment (nSOFA; Berka et al., 2022), and Prognostic Respiratory Intensity Scoring Metric (PRISM; Nance et al., 2025) scores.

For HeRO, three metrics were considered: the value closest to the MRI date, the value closest to birth, and the maximum score recorded during hospitalization as an index of peak illness burden. For nSOFA, single time-point values showed minimal variability across participants (97.37% zero values); therefore, only maximum nSOFA scores were analyzed. PRISM scores reflect cumulative illness severity during hospitalization and were therefore analyzed as single summary values.

Our preregistration included the hypothesis that lower fiber density in motor tracts would be associated with delayed gross motor outcomes. However, charted motor outcome measures were sparse in the electronic health record, and as such, this hypothesis could not be evaluated with the current dataset.

Associations between illness severity and imaging measures were tested using linear mixed-effects models including illness severity scores as fixed effects. Additional models examined interactions between illness severity and tract classification for fiber density analyses, and between illness severity and tissue type for tissue signal fraction analyses. Multiple comparisons correction was performed using the Benjamini–Hochberg false discovery rate (FDR). FDR-corrected *p*-values are reported as *q*-values.

## 3 Results

### 3.1 Participant characteristics

The analytic sample included 43 neonates (27 female). The majority of participants were White (67%), with 16% identifying as Black or African American, and 16% as Other, multiracial, or Hispanic/Latino. Most infants were singleton births (65%), with 28% twins and 7% triplets. Mean gestational age (GA) at birth was 201.8 ± 31.0 days (~28.8 ± 4.4 weeks). The majority of participants (53.49%) were classified as Extremely Preterm (< 28 weeks' GA), with 23.26% born Very Preterm (28–32 weeks' GA), 16.28% born Moderate to Late Preterm (32–37 weeks' GA), and 6.98% born Early Term (37–40 weeks' GA). Mean postmenstrual age at MRI was 282.6 ± 30.7 days (~40.4 ± 4.4 weeks). Delivery was predominantly by Cesarean section (56%), with 16% born vaginally; delivery method was not reported for the remaining participants. A summary of clinical MRI findings is provided in Table 1.

**Table 1:** Summary of clinical MRI findings.

Category	*N*	%
Normal MRI/no intracranial abnormality	18	41.9
Prior hemorrhage/germinal matrix hemorrhage/microhemorrhage	11	25.6
White matter/hypoxic-ischemic injury	4	9.3
Prominent extra-axial or subarachnoid spaces	6	14.0
Ventriculomegaly/hydrocephalus	3	7.0
Major congenital brain malformation	3	7.0
Reduced brain volume/diffuse volume loss	3	7.0
Subdural hemorrhage	3	7.0

### 3.2 White matter microstructure derived from clinical-quality scans reproduces expected anatomical and developmental patterns

**Figure 6:**
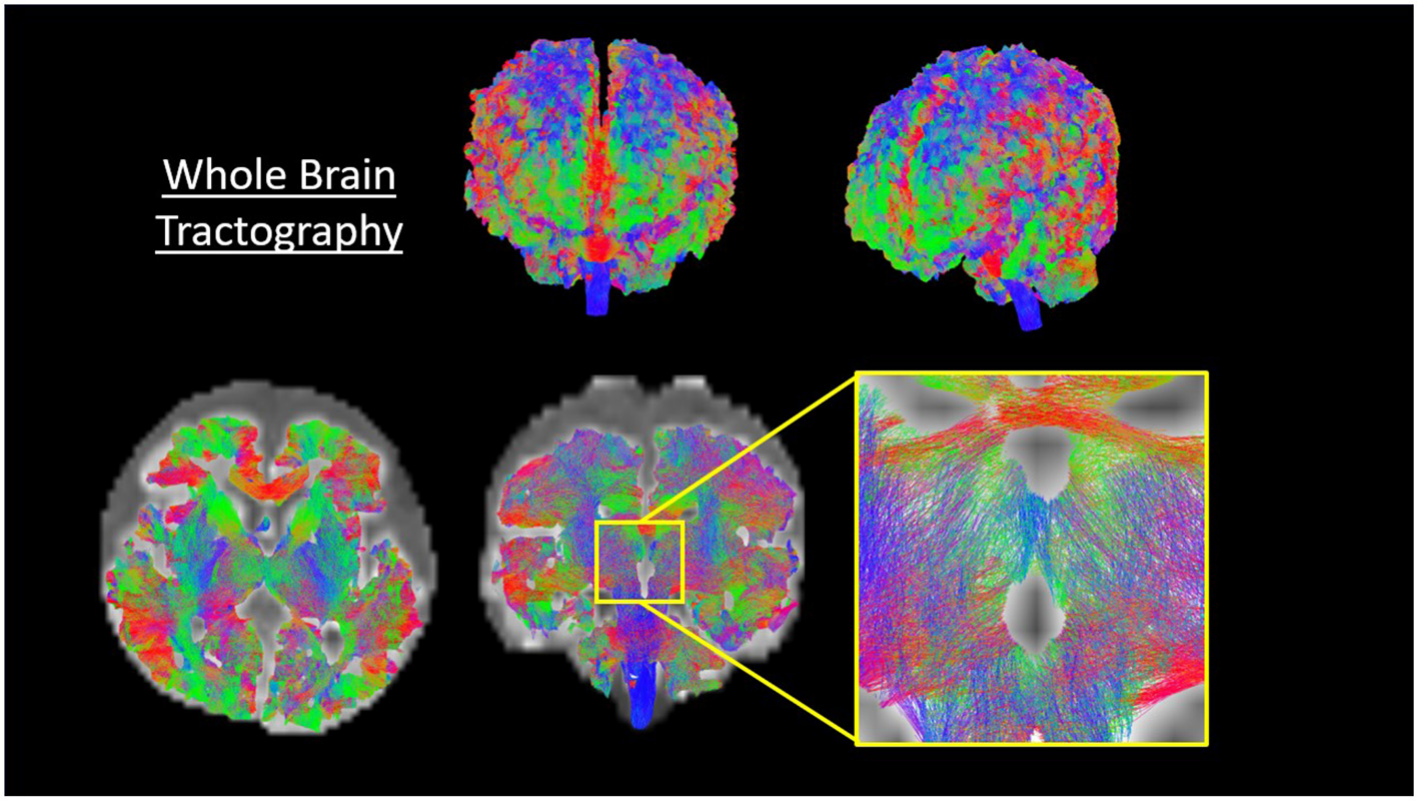
Representative whole brain tractogram. Using probabilistic fiber tracting and established algorithms ACT, iFOD2, and SIFT we were able to generate tracts with similar sensitivity, coverage, and complexity to adult subjects. Crossing fibers were successfully detected and tracked (see insert) while using SIFT permitted evaluation of tracts using underlying axonal anatomical constraints. Resulting tractograms contained 1 million tracts for each participant, permitting connectome-based strength and connectivity comparisons.

**Figure 7:**
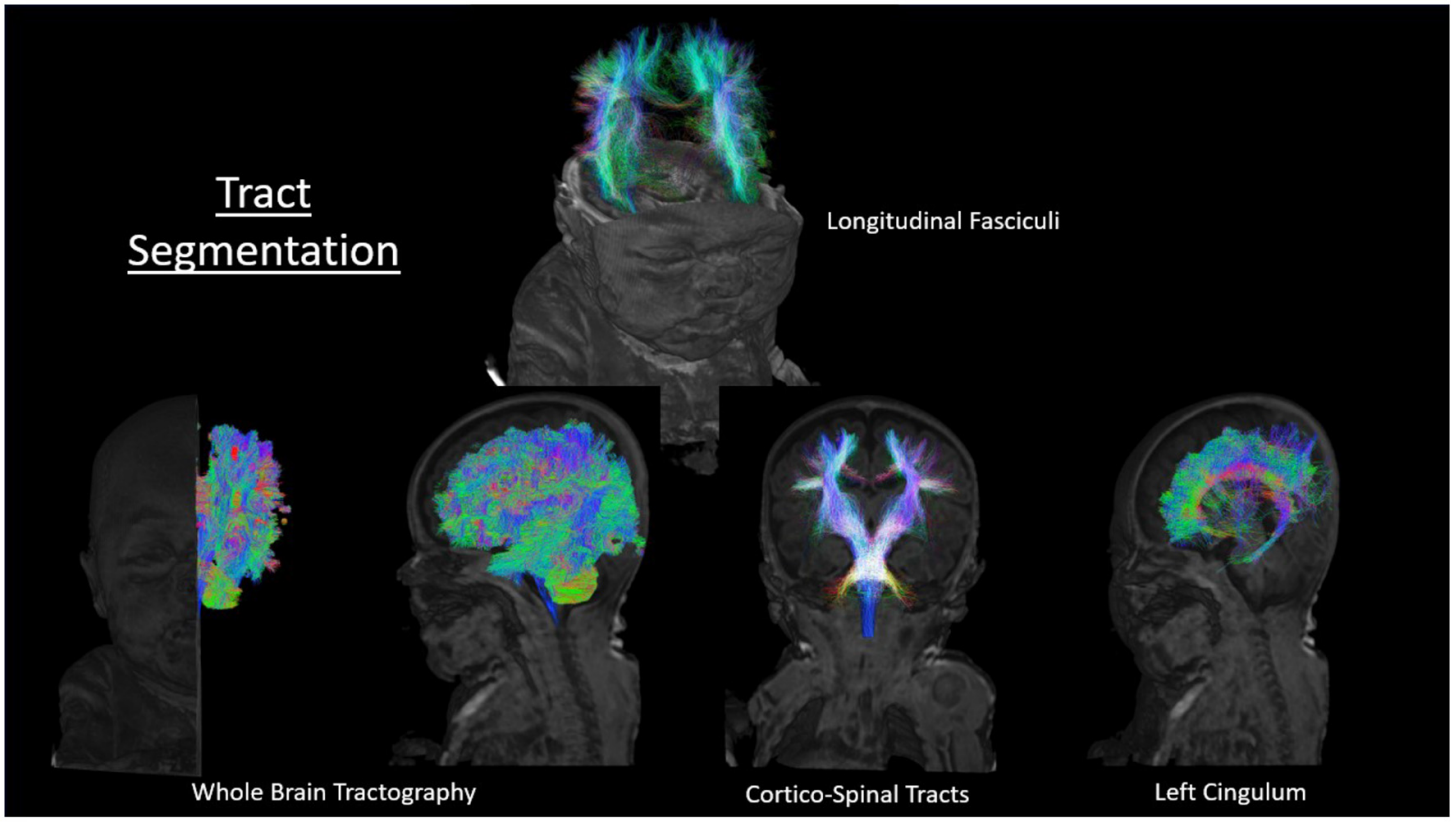
Extraction of major white matter tracts, as extracted via TractSeg, in representative data from the cohort. Combining whole-brain tractograms and TractSeg extracted voxels permits the illustration and quantification of major white matter structures, including longitudinal fasciculi, cortico-spinal tracts, and the cingulum. In addition to demonstrating anatomical accuracy for the entire pipeline, this work potentially opens the door to tract-based spatial statistics and tract-specific measures in the neonatal population.

**Figure 8:**
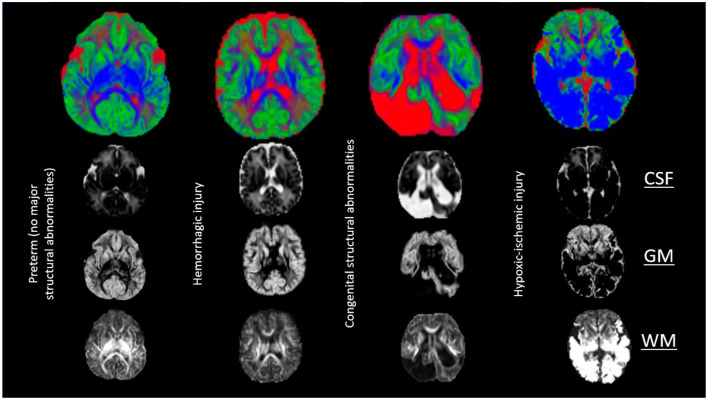
Illustration of various pathologies within the neonatal population and their representation by 3T-CSD microstructural metrics. Illustrative examples of microstructural signal patterns observed across the cohort, including cases with hemorrhage, ventricular enlargement, and ischemic injury.

Our diffusion modeling pipeline produced anatomically coherent whole-brain tractograms from clinically acquired diffusion data. Probabilistic tractography generated dense white matter reconstructions across the neonatal brain, including successful detection and tracking of crossing fibers (Figure 6). Segmentation of these tractograms using TractSeg further enabled identification of major projection and association pathways, including corticospinal tracts, longitudinal fasciculi, and the cingulum (Figure 7), demonstrating that clinically-acquired diffusion scans can support tract-specific analyses using this framework. In addition to enabling tractography and segmentation, the resulting 3T-CSD microstructure maps provided interpretable tissue-specific signatures that could reflect common neonatal neuropathology (Figure 8), illustrating the potential clinical utility of this approach for characterizing brain microstructure in vulnerable populations.

Summary statistics for fiber density by tract and tissue signal fraction by subcortical structure are provided in Table 2. Fiber density differed between tract classes in the expected pattern. Early-maturing tracts showed higher mean fiber density (0.67 ± 0.25) than later-developing association tracts (0.45 ± 0.16), consistent with neonatal white matter maturation patterns. A Welch two-sample *t*-test confirmed this difference was highly significant (*t* = 20.91, *p* < 0.001, 95% CI [0.20, 0.24]).

**Table 2:** Fiber density and tissue signal fraction summary statistics.

Classification	Tract/structure	*N*	Mean	SD
Fiber density in early-emerging tracts	Corpus callosum: Isthmus	40	0.56	0.14
	Corpus callosum: Posterior midbody	41	0.59	0.39
	Corpus callosum: Splenium	35	0.51	0.07
	Cortico-spinal tract	39	0.88	0.13
	Fronto-pontine tract	40	0.76	0.22
	Middle cerebellar peduncle	40	0.77	0.17
	Optic radiation	39	0.55	0.12
	Parieto-occipital pontine	40	0.81	0.18
	Superior-cerebellar peduncle	37	0.8	0.15
	Thalamo-occipital tract	38	0.53	0.11
	Thalamo-parietal tract	43	0.64	0.26
	Thalamo-precentral tract	43	0.54	0.37
Fiber density in later-developing tracts	Anterior thalamic radiation	38	0.39	0.1
	Arcuate fascicle	38	0.46	0.11
	Cingulum	38	0.44	0.11
	Corpus callosum: Genu	34	0.36	0.06
	Inferior longitudinal fascicle	42	0.39	0.31
	Inferior occipito-frontal fascicle	36	0.45	0.09
	Middle longitudinal fascicle	36	0.45	0.09
	Striato-occipital tract	37	0.51	0.1
	Striato-parietal tract	40	0.63	0.15
	Striato-prefrontal tract	36	0.37	0.08
	Thalamo-prefrontal tract	39	0.42	0.11
Subcortical structure tissue fraction	Accumbens	35	0.26	0.25
	Amygdala	35	0.2	0.29
	Caudate	35	0.33	0.28
	Hippocampus	35	0.22	0.25
	Pallidum	35	0.31	0.36
	Putamen	35	0.34	0.31
	Thalamus	35	0.34	0.31

Subcortical tissue signal fractions also showed the expected distribution across tissue types. A repeated-measures ANOVA revealed a significant effect of tissue type on mean signal fraction [*F*_(2, 93)_ = 125.11, *p* < 0.001, generalized η^2^ = 0.729]. Bonferroni-corrected paired comparisons indicated that white matter fractions (*M* = 0.536) were higher than gray matter (*M* = 0.299) and CSF (*M* = 0.041), and gray matter fractions were higher than CSF (all *p*s < 0.001).

### 3.3 Developmental increases in fiber density reveal tract-specific maturation patterns

We used a linear mixed-effects model to examine associations between postmenstrual age and tract class on fiber density. Fiber density increased significantly with postmenstrual age (β = 0.0018, SE = 0.00044, *p* < 0.001) and with GA (β = 0.0014, SE = 0.00041, *p* = 0.002). Early-maturing tracts showed significantly higher fiber density than later-developing association tracts (β = −0.224, SE = 0.0095, *p* < 0.001). Sex was not associated with fiber density (*p* = 0.535).

**Figure 9:**
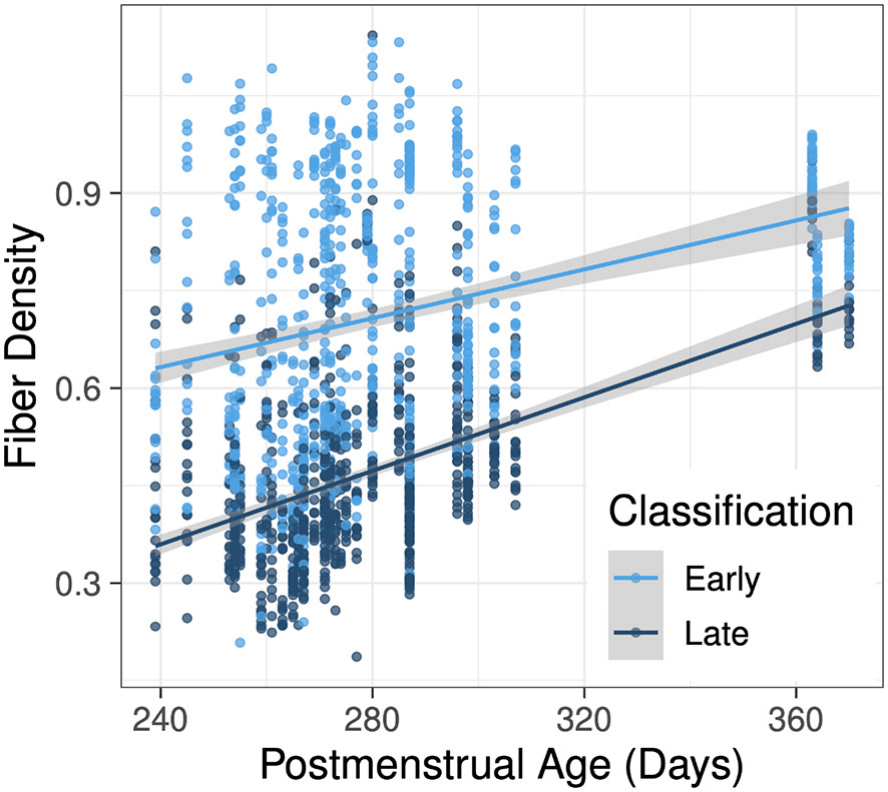
Interaction between postmenstrual age and tract class on fiber density. Fiber density was higher in early-emerging tracts, and increased with postmenstrual age in both early-maturing and later-developing white matter tracts at different rates. Specifically, later-developing association tracts exhibited a slightly steeper increase in fiber density with age compared to early-maturing tracts.

There was also a significant interaction between postmenstrual age and tract class (β = 0.0011, SE = 0.0003, *p* = 0.001), indicating that the association between postmenstrual age and signal fraction differed between tract classes. Specifically, later-developing tracts showed a slightly steeper increase in fiber density with age compared to early-maturing tracts (Figure 9). Sensitivity analyses testing more lenient (tract zero values > 40%; subject zero values > 35%) and stringent (tract zero values > 60%; subject zero values > 15%) exclusion criteria yielded consistent patterns of results (Supplementary Table S2).

To evaluate robustness of these findings in the younger subset of infants, a parallel model showed similar developmental effects (Supplementary Figure S1). Fiber density increased with postmenstrual age (β = 0.0030, SE = 0.00089, *p* = 0.002) and GA (β = 0.0013, SE = 0.00045, *p* = 0.006), and was higher in early-maturing tracts (β = −0.239, SE = 0.011, *p* < 0.001). However, the interaction between postmenstrual age and tract class did not reach statistical significance in this age-restricted subset (*p* = 0.577). Sex was again not associated with fiber density (*p* = 0.414).

### 3.4 Tissue signal fractions differ across brain compositions and show distinct developmental trajectories in white and gray matter

**Figure 10:**
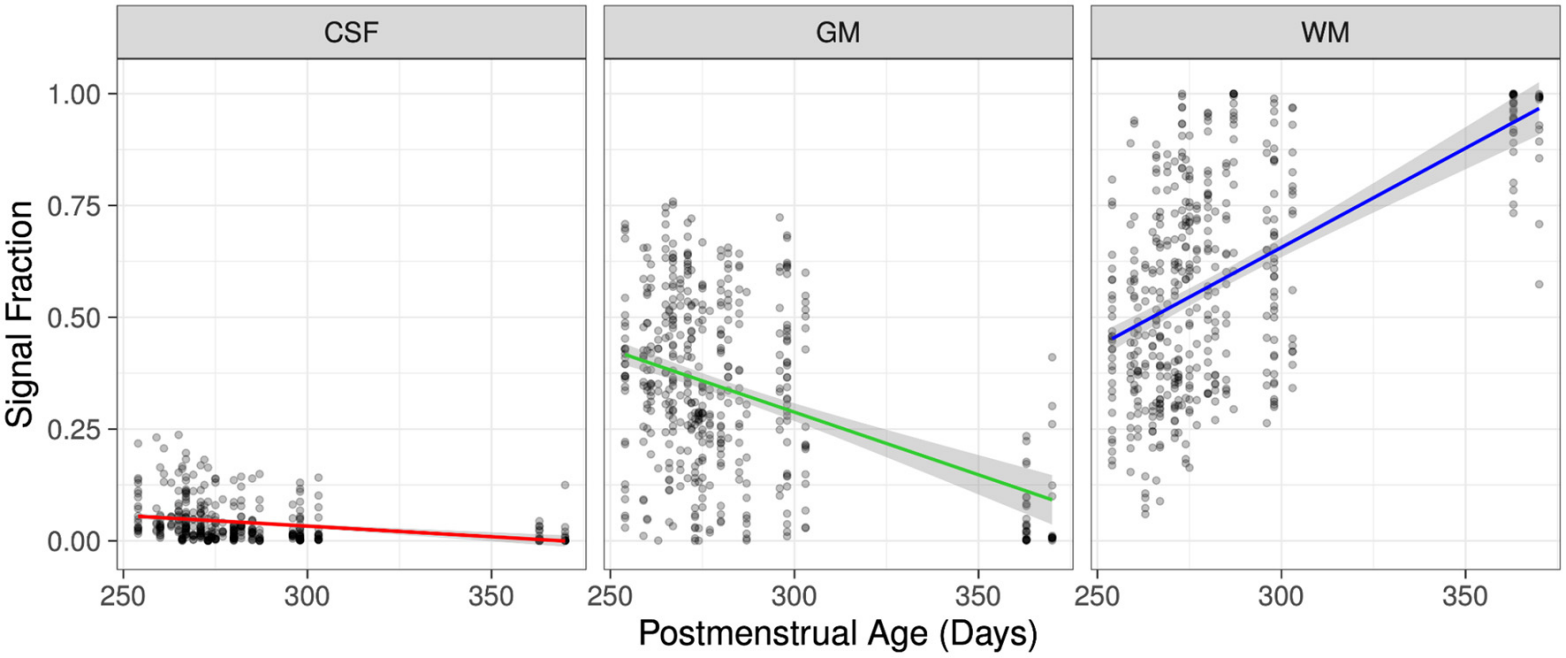
Developmental trajectories of signal fraction differed by tissue type. Signal fraction increased with postmenstrual age in white matter (WM), decreased in gray matter (GM), and demonstrated slight, albeit non-significant, decreases in cerebrospinal fluid (CSF), indicating distinct maturation patterns across brain tissues. Red, CSF-like; green, gray matter-like; and blue, white matter-like signal fraction.

**Figure 11:**
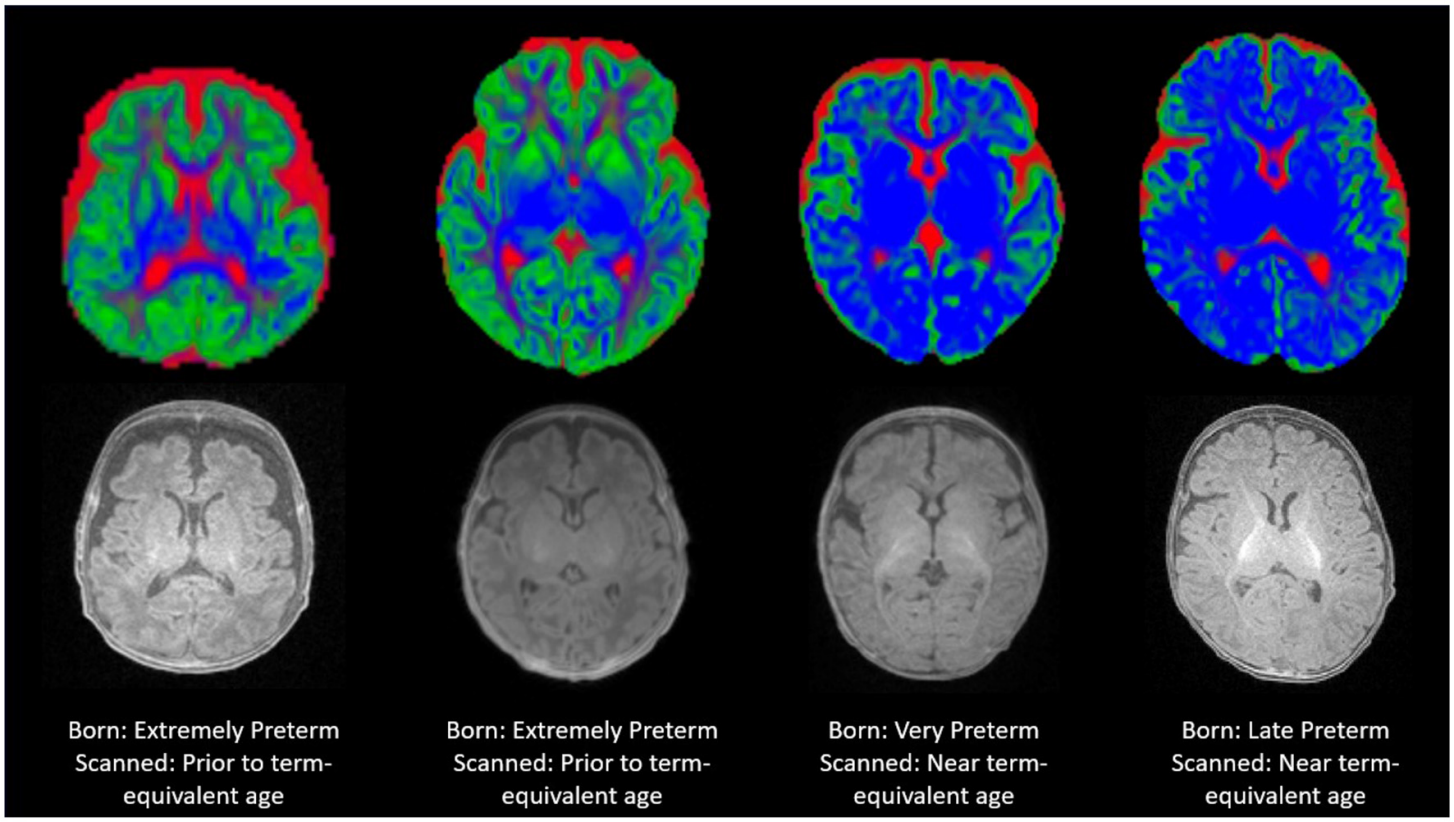
Comparison of age-related changes in brain composition of 3T-CSD maps. **(Top row)** red, CSF-like; green, gray matter-like; and blue, white matter-like; **(bottom row)** T1-weighted MPRAGE images. Despite relatively similar T1-weighted images and normal clinical reports, the degree of change in 3T-CSD metrics is apparent, with a general increase in white matter-like signal fraction and a decrease in gray matter-like signal fraction across the entire cortex and subcortical gray matter. Development of the frontal cortex is also highly apparent.

We used a linear mixed-effects model to examine associations between postmenstrual age and tissue type on signal fraction. Signal fraction differed significantly across tissue classes (Figure 10). Relative to CSF, signal fraction was substantially higher in gray matter (β = 0.296, SE = 0.013, *p* < 0.001) and white matter (β = 0.562, SE = 0.013, *p* < 0.001). Although there was no overall main effect of postmenstrual age (*p* = 0.348), the association between postmenstrual age and signal fraction differed by tissue type. Specifically, signal fraction increased with postmenstrual age in white matter (β = 0.0046, SE = 0.00043, *p* < 0.001) but decreased in gray matter (β = −0.0028, SE = 0.00043, *p* < 0.001). GA (*p* = 0.659) and sex (*p* = 0.798) were not significantly associated with signal fraction. 3T-CSD maps illustrating age-related changes in brain composition can be seen in Figure 11. Sensitivity analyses demonstrated that these results were robust to variation in exclusion thresholds (Supplementary Table S3).

Analyses restricted to the younger subset yielded a similar pattern (Supplementary Figure S2). Signal fraction again differed significantly across tissue classes (both *p* < 0.001). The interaction between postmenstrual age and white matter remained significant (β = 0.0064, SE = 0.0011, *p* < 0.001), indicating increasing signal fraction with age in white matter. In contrast, the postmenstrual age-by–gray matter interaction was not significant in this subset (*p* = 0.439). Neither GA (*p* = 0.494) nor sex (*p* = 0.610) was associated with signal fraction.

### 3.5 Illness severity shows minimal associations with tissue signal fractions

Exploratory analyses examined whether illness severity was associated with fiber density and tissue signal fraction. Across models including HeRO scores (closest to MRI, at birth, and maximum during hospitalization), nSOFA maximum scores, and PRISM scores, no significant main effects of illness severity were observed for signal fraction after controlling for postmenstrual age, GA at birth, and sex (all *p*s > 0.313). Illness severity measures also did not interact with tract classification in fiber density models (all *p*s > 0.133).

**Figure 12:**
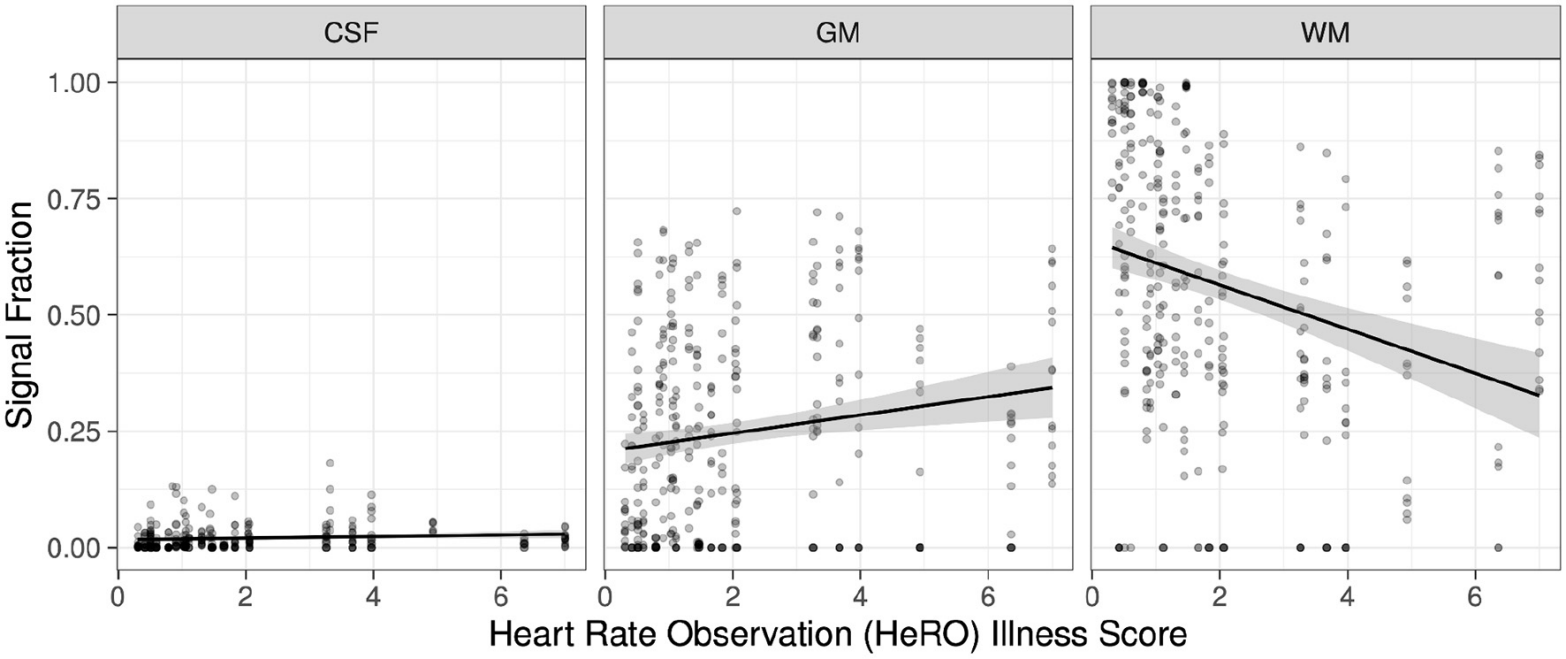
Associations between illness severity and tissue signal fraction. A significant interaction between Heart Rate Observation (HeRO) illness score at birth and tissue type indicated differential relationships across brain tissues. Higher HeRO scores at birth were associated with increased gray matter (GM) signal fraction and decreased white matter signal fraction (WM). There was no association with cerebral spinal fluid (CSF) signal fraction.

In tissue signal fraction models, a significant interaction between HeRO score at birth and tissue type was observed, indicating differential associations across tissues (Figure 12). Specifically, higher HeRO scores at birth were associated with higher gray matter signal fraction (β = 0.023, SE = 0.009, *q* = 0.019) and lower white matter signal fraction (β = −0.045, SE = 0.0087, *q* < 0.001). In contrast, HeRO scores closest to MRI (β = −0.101, SE = 0.036, *q* = 0.018) and PRISM (β = −0.0002, SE < 0.0001, *q* = 0.018) showed a significant association only with white matter signal fraction; associations with gray matter signal fraction did not survive multiple comparisons correction (*q*s > 0.178). Interactions between tissue type and maximum HeRO scores and nSOFA were not significant (all interaction *q*s > 0.187).

## 4 Discussion

This study demonstrates that fixel-based measures of white matter microstructure can be reliably derived from clinically-acquired neonatal diffusion MRI. Using a constrained spherical deconvolution framework adapted for low-resolution clinical scans, we generated fiber density and tissue signal fraction maps that supported tract-specific analyses and subcortical tissue segmentation across the neonatal brain. These measures reproduced expected patterns of early white matter maturation, including higher fiber density in early-developing projection tracts and developmental increases in white matter signal fraction with postmenstrual age. Together, these findings show that clinically-acquired diffusion MRI, even when acquired with lower spatial and angular resolution, can support biologically meaningful microstructural analyses, expanding the potential for advanced diffusion modeling in neonatal and other clinical populations.

Consistent with prior studies of neonatal brain development (Ball et al., 2013; Braga et al., 2015), we observe clear tract-specific maturation patterns. Fiber density was higher in early-maturing projection tracts than in later-developing association pathways, and tissue signal fraction increased with postmenstrual age in white matter while gray matter showed distinct developmental trajectories. Importantly, although tract-level differences aligned with established maturation hierarchies, these comparisons were based on *a priori* classifications and therefore serve as consistency checks; the primary evidence for developmental sensitivity arises from continuous associations with postmenstrual age. These findings indicate that biologically meaningful developmental signals can be detected even in clinically acquired diffusion scans of preterm infants.

Exploratory analyses examined associations between clinical illness severity and microstructural measures. Notably, at birth, higher HeRO scores, a measure derived from continuous heart rate monitoring that indexes autonomic instability and risk for neonatal sepsis, were associated with higher gray matter fraction and lower white matter fraction, indicating differential associations across tissue types. In contrast, HeRO scores closest to MRI and PRISM scores were associated only with lower white matter signal fraction, and these effects did not extend to gray matter after correction for multiple comparisons. Maximum HeRO scores during hospitalization and nSOFA were not significantly associated with tissue signal fractions. The finding that HeRO at birth showed the most consistent and tissue-differentiated pattern of associations suggests that early physiological instability may exert a cascading influence on brain development, highlighting the importance of early insults in shaping subsequent microstructural trajectories (Mathur et al., 2010). Associations with HeRO closest to MRI and PRISM may reflect more contemporaneous or cumulative illness burden, but their restriction to white matter and reduced consistency across tissues suggests a less specific relationship with underlying microstructural organization. However, these findings should be considered exploratory and interpreted with caution. Additionally, the selected cohort was recruited exclusively for preterm birth and available imaging, resulting in substantial clinical heterogeneity; studies in more targeted populations may help clarify the specificity of these associations.

A key contribution of this work is the demonstration that clinically-acquired MRI scans, which often have lower spatial and angular resolution than research-grade acquisitions, can nevertheless yield meaningful microstructural information when analyzed with advanced multi-compartment modeling techniques. The approach described in this study does not require any specialized acquisitions, relies entirely on publicly-available software, and does not even necessitate the co-acquisition of a T1-weighted image. The resulting tractography, tract segmentation, and microstructural metrics successfully identified and segmented major white matter tracts and reproduced established patterns of neonatal white matter organization and maturation. These findings suggest that large archives of clinically acquired neonatal diffusion scans may represent an underutilized resource for studying early brain development and the effects of perinatal illness. This approach therefore increases the feasibility of studying vulnerable neonatal populations without requiring research-dedicated imaging sessions, opens the possibility of leveraging large retrospective clinical imaging datasets to investigate early brain development and identify neuroimaging markers of neonatal risk, and provides a practical framework for broader implementation in hospital settings.

In previously published studies, our group has highlighted the ability of diffusion microstructure to detect functionally significant differences in autism spectrum disorder (ASD; Newman et al., 2024, 2025b; Ressa et al., 2024; Coleman et al., 2025). Like several other childhood behavioral disorders, ASD is typically diagnosed after behavioral differences manifest in early childhood. Calculation of microstructure metrics in adolescents who go on to receive ASD diagnoses may identify early signs or patterns that improve detection and allow for earlier intervention. This same approach could be used to benefit attention deficit hyper-activity disorder (ADHD), and other early manifesting child behavioral disorders.

### 4.1 Limitations and future directions

Several limitations should be noted. The sample size was modest, and participants displayed heterogeneous brain injury patterns, limiting statistical power and generalizability. Imaging was acquired opportunistically during clinical care, leading to variability in scan quality. Additionally, although advanced modeling approaches improve sensitivity, inherent limitations of diffusion MRI, such as sensitivity to motion and partial volume effects, remain, particularly in unmyelinated neonatal tissue (Dubois et al., 2021). The lack of a unified gold standard for quantifying myelin, combined with the clinical nature of the dataset, limits direct validation and restricts conclusions to consistency across tissue compartments and qualitative tractography accuracy. Finally, our cross-sectional design does not allow us to make inferences about the long-term functional implications of observed microstructural differences. Future work should leverage longitudinal follow-up to examine how early microstructural differences predict later neurodevelopmental outcomes. Methodologically, extending these approaches to higher-resolution acquisitions, and combining diffusion metrics with complementary imaging modalities, could further refine the sensitivity and specificity of neonatal microstructural assessment.

### 4.2 Conclusion

Overall, these findings underscore the feasibility and utility of extracting rich microstructural information from routine clinical scans, opening avenues for studying early brain development in preterm infants at scale.

## Data Availability

The raw data supporting the conclusions of this article will be made available by the authors, without undue reservation.
