# Supplementary material for: A novel fixel-based approach for resolving neonatal white matter microstructure from clinical diffusion MRI
Source: Front Neurosci. 2026 Jun 5;20:1830684. doi: 10.3389/fnins.2026.1830684 (PMC13279704; doi:10.3389/fnins.2026.1830684)
Supplement: Supplementary file 1 [file Data_Sheet_1.docx]

#
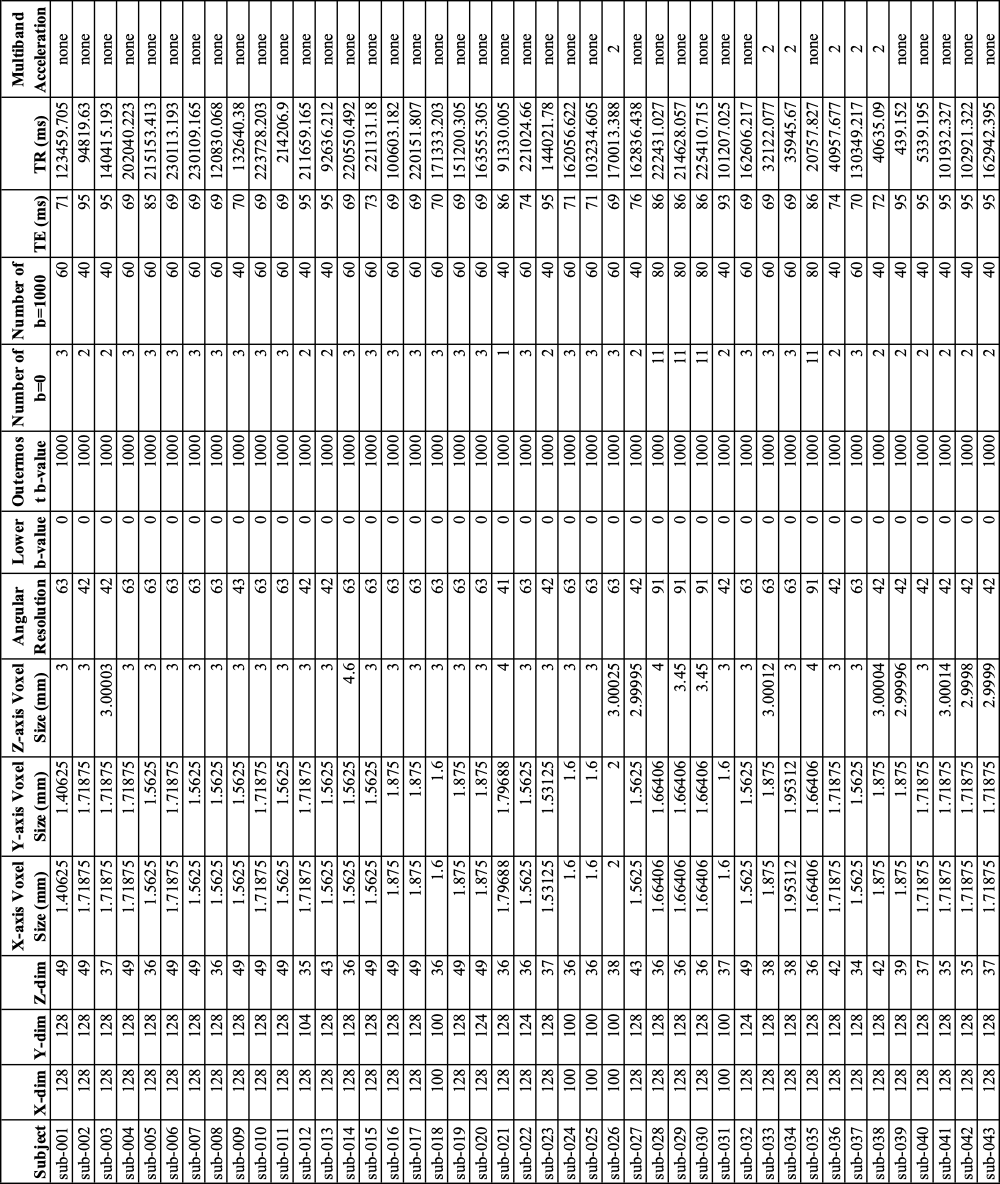


**Table S1. Acquisition parameters for each scan.**

|  |  | **Original** | **Lenient** | **Strict** |
| --- | --- | --- | --- | --- |
|  | Excluded n | 6 | 3 | 30 |
|  | Tract zero value threshold | 50% | 40% | 60% |
|  | Subject zero value threshold | 25% | 35% | 15% |
| **Fixed Effects** | PMA | 0.002 (0.000) *** | 0.002 (0.000) *** | 0.002 (0.001) ** |
|  | Tract Classification | -0.224 (0.009) *** | -0.208 (0.011) *** | -0.140 (0.009) *** |
|  | GA | 0.001 (0.000) ** | 0.001 (0.000) ** | 0.003 (0.001) *** |
|  | Sex | 0.016 (0.026) | 0.024 (0.027) | -0.064 (0.055) |
|  | PMA:Tract Classification | 0.001 (0.000) ** | 0.001 (0.000) ** | 0.001 (0.000) ** |

**Table S2. Sensitivity analyses of fiber density using alternative tract- and subject-level exclusion thresholds.** For each fixed effect, we present estimates (β) with standard errors in parentheses. Astericts denote statistical significance (*** *p* < .001; ** *p* < .01). Abbreviations: PMA, postmenstrual age; GA, gestational age.

|  |  | **Original** | **Lenient** | **Strict** |
| --- | --- | --- | --- | --- |
|  | Excluded n | 3 | 0 | 16 |
|  | Tract zero value threshold | 50% | 40% | 60% |
|  | Subject zero value threshold | 25% | 35% | 15% |
| **Fixed Effects** | PMA | 0.000 (0.000) | -0.001 (0.000) | 0.000 (0.000) |
|  | GM | 0.296 (0.013) *** | 0.307 (0.014) *** | 0.284 (0.015) *** |
|  | WM | 0.562 (0.013) *** | 0.555 (0.014) *** | 0.571 (0.015) *** |
|  | GA | 0.000 (0.000) | 0.000 (0.000) | 0.000 (0.000) |
|  | Sex | -0.003 (0.013) | -0.004 (0.012) | -0.003 (0.017) |
|  | PMA:GM | -0.003 (0.000) *** | -0.003 (0.000) *** | -0.003 (0.000) *** |
|  | PMA:WM | 0.005 (0.000) *** | 0.005 (0.000) *** | 0.005 (0.000) *** |

**Table S3. Sensitivity analyses of tissue signal fractions using alternative tract- and subject-level exclusion thresholds.** For each fixed effect, we present estimates (β) with standard errors in parentheses. Astericts denote statistical significance (*** *p* < .001). Abbreviations: PMA, postmenstrual age; GM, grey matter; WM, white matter; GA, gestational age.


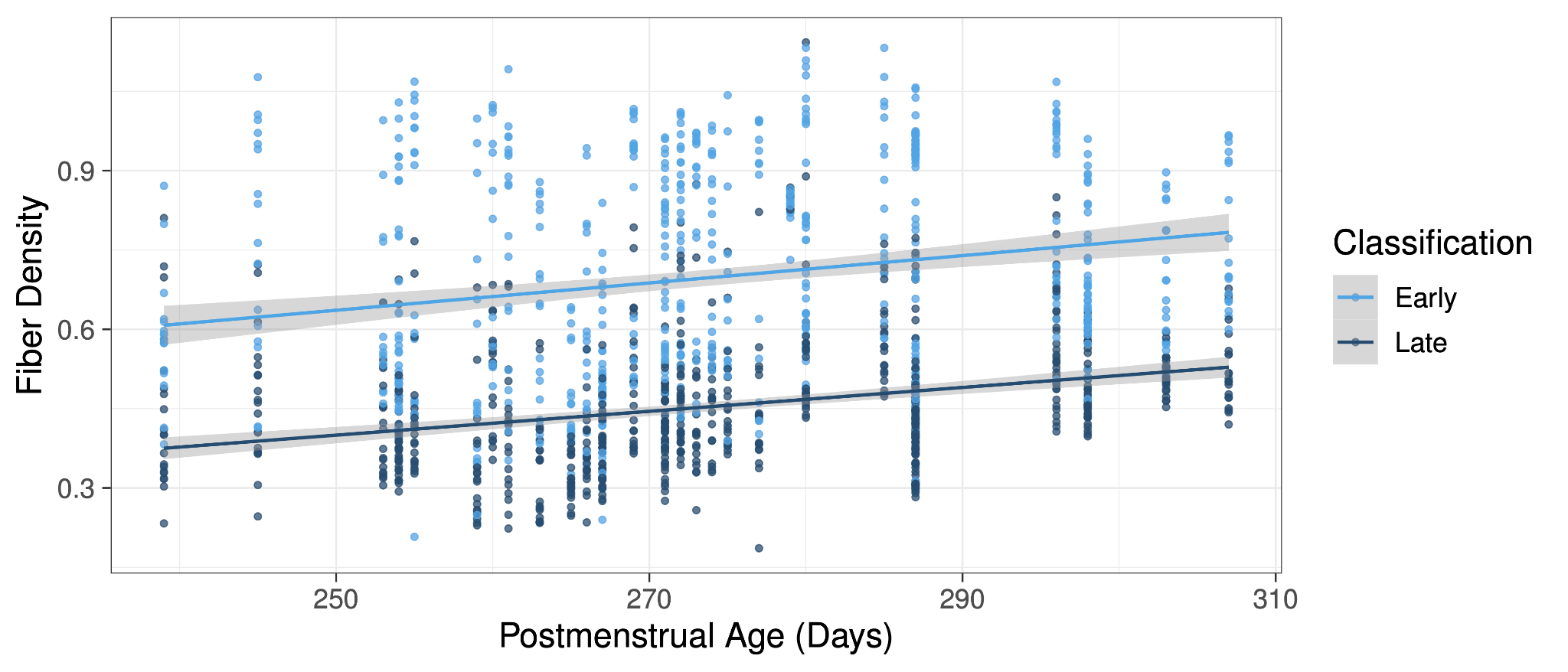


**Figure S1. Association between postmenstrual age and tract class on fiber density in an age-restricted sub-sample.** In a sub-sample of younger infants (postmenstrual age < 360 days), fiber density was higher in early-emerging tracts and increased with postmenstrual age in both early-maturing and later-developing white matter tracts. However, the rate of development of these tracts did not differ in this age-restricted sub-sample, indicating that differences in developmental trajectories between tract classes emerge across a broader developmental window.


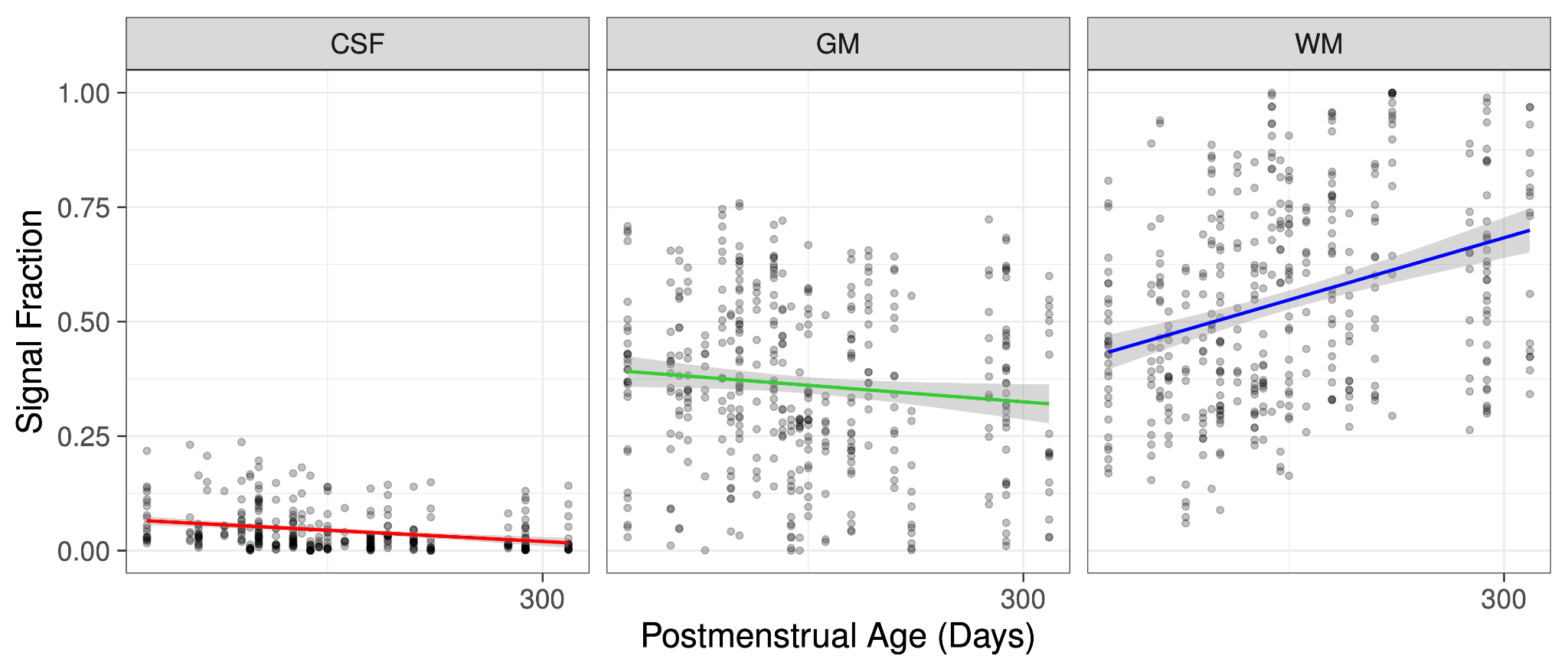


**Figure S2.** **Associations between postmenstrual age and tissue signal fraction in an age-restricted sub-sample.** In a sub-sample of younger infants (postmenstrual age < 360 days), the significant positive association between postmenstrual age and signal fraction in white matter (WM) remained. However, in this age-restricted sub-sample, the negative association with both grey matter (GM) and cerebrospinal fluid (CSF) were non-significant, indicating that the developmental increase observed in WM signal fraction is robust even within a narrower age range. Red=CSF-like, green=grey matter-like, and blue=white matter-like signal fraction.
